# The *Mycobacterium tuberculosis*Uganda II family and resistance to first-line anti-tuberculosis drugs in Uganda

**DOI:** 10.1186/s12879-014-0703-0

**Published:** 2014-12-19

**Authors:** Nicholas Ezati, Deus Lukoye, Eddie M Wampande, Kenneth Musisi, George W Kasule, Frank GJ Cobelens, David P Kateete, Moses L Joloba

**Affiliations:** National Tuberculosis and Leprosy Program, Kampala, Uganda; National Tuberculosis Reference Laboratory (NTRL), Kampala, Uganda; Department of Medical Microbiology, Makerere University College of Health Sciences, Kampala, Uganda; Department of Global health and Amsterdam Institute for Global Heath and Development, Academic Medical Center, Amsterdam, The Netherlands KNCV Tuberculosis Foundation, The Hague, The Netherlands; Department of Bio-molecular Resources and Biolab Sciences, College of Veterinary Medicine, Animal Resources and Bio Security, Makerere University, Kampala, Uganda

## Abstract

**Background:**

The global increase in the burden of multidrug-resistant tuberculosis (MDR-TB) underscores an urgent need for data on factors involved in generation and spread of TB drug resistance. We performed molecular analyses on a representative sample of *Mycobacterium tuberculosis* (MTB) isolates. Basing on findings of the molecular epidemiological study in Kampala, we hypothesized that the predominant MTB strain lineage in Uganda is negatively associated with anti-TB drug resistance and we set out to test this hypothesis.

**Methods:**

We extracted DNA from mycobacterial isolates collected from smear-positive TB patients in the national TB drug resistance survey and carried out IS6110-PCR. To identify MTB lineages/sub lineages RT-PCR SNP was performed using specific primers and hybridization probes and the ‘melting curve’ analysis was done to distinguish the Uganda II family from other MTB families. The primary outcome was the distribution of the Uganda II family and its associations with anti-TB drug resistance and HIV infection.

**Results:**

Out of the 1537 patients enrolled, MTB isolates for 1001 patients were available for SNP analysis for identification of Uganda II family, of which 973 (97%) had conclusive RT-PCR results. Of these 422 (43.4%) were of the Uganda II family, mostly distributed in the *south west* zone (55.0%; OR = 4.6 for comparison with other zones; 95% CI 2.83-7.57; p < 0.001) but occurred in each of the other seven geographic zones at varying levels. Compared to the Uganda II family, other genotypes as a group were more likely to be resistant to any anti-TB drug (OR^adj^ =2.9; 95% CI 1.63-5.06; p = 0.001) or MDR (OR^adj^ 4.9; 95% CI, 1.15-20.60; p = 0.032), even after adjusting for geographic zone, patient category, sex, residence and HIV status. It was commonest in the 25–34 year age group 159/330 (48.2%). No association was observed between Uganda II family and HIV infection.

**Conclusion:**

The Uganda II family is a major cause of morbidity due to TB in all NTLP zones in Uganda. It is less likely to be resistant to anti-TB drugs than other MTB strain lineages.

**Electronic supplementary material:**

The online version of this article (doi:10.1186/s12879-014-0703-0) contains supplementary material, which is available to authorized users.

## Background

Tuberculosis (TB) remains one of the major causes of morbidity and mortality, estimated to affect a third of the population worldwide. Through the past two to three decades TB incidence and mortality have markedly increased as a result of HIV [1], and an estimated half million multidrug resistant (MDR) TB cases exist across the world. The association between MDR-TB and HIV infection in sub-Saharan Africa (SSA) remains a controversial subject with no association documented in most settings [2]-[5], although some studies continue to report an association between these two disease epidemics at individual level [6]-[9]. The increasing global burden of MDR-TB underscores the urgent need for pathogen and host data on factors involved in generation and spread of drug resistant strains, so as to facilitate focussed interventions by the national programs for its control [10], especially in low resource and high burden TB, HIV and TB/HIV co-infection settings such as SSA.

Uganda is still counted among the 22 high TB burden countries worldwide with an estimated 293 per 100,000 incident cases of all TB forms in 2010 [11]. It has a high HIV prevalence, currently estimated at 7.3% among the 15–49 years age group in the general population (AIS 2011), with a TB/HIV co-infection rate of 54% in the 2010 TB cohort [11]. Results from the national drug resistance survey show the prevalence of MDR-TB to be low; 2.3% among sputum smear-positive new and previously treated TB patients combined [5].

Molecular epidemiological studies are useful for understanding key aspects of TB control including transmission, potential to cause disease outbreaks, and resistance to anti-TB drugs [8],[12],[13], among others. Molecular techniques commonly used in these studies [14],[15] include spacer oligonucleotide typing (spoligotyping), region of difference (RD) analysis, mycobacteria insertion repetitive units (MIRU) analysis, insertion sequence 6110 (IS6110) restriction fragment length polymorphism (RFLP) [13],[14], single nucleotide polymorphisms (SNP) and whole genome sequencing now the gold standard. Among these, SNPs represent reliable markers for lineage classification of *Mycobacterium tuberculosis complex* [16],[17].

The Uganda II genotype is a sub lineage of the MTB lineage 4, the Euro- American lineage which is defined by RD 724 deletion [18]. By spoligotyping, it is defined by deletion of spacer 33–36 and spacer 40 [19]. Using the highly sensitive and specific SNP technique, the Uganda family is further subdivided into the Uganda I and Uganda II sub-types which contribute 15% and 85% respectively to this family in Uganda, although these studies were limited with respect to geographical coverage [20],[21]. Earlier studies have identified the T2 family in general as an important MTB genotype in specific localities in Uganda [19],[20] and shown a negative association with drug resistance in a limited geographic setting [22]. This finding is potentially relevant for understanding the role of genotype diversity in the epidemiology of TB drug resistance in Uganda and elsewhere in East Africa, but calls for confirmation in a geographically more diverse dataset, among others to establish whether unrelated regional differences in genotype distribution could confound this association. Our hypothesis in this study therefore was that the Uganda II MTB subtype is the major cause of morbidity due to tuberculosis in Uganda and is negatively associated with anti-TB drug resistance, and we aimed at testing this hypothesis in a larger and geographically representative data set.

## Methods

### Sampling and sample size estimation

**Figure 1:**
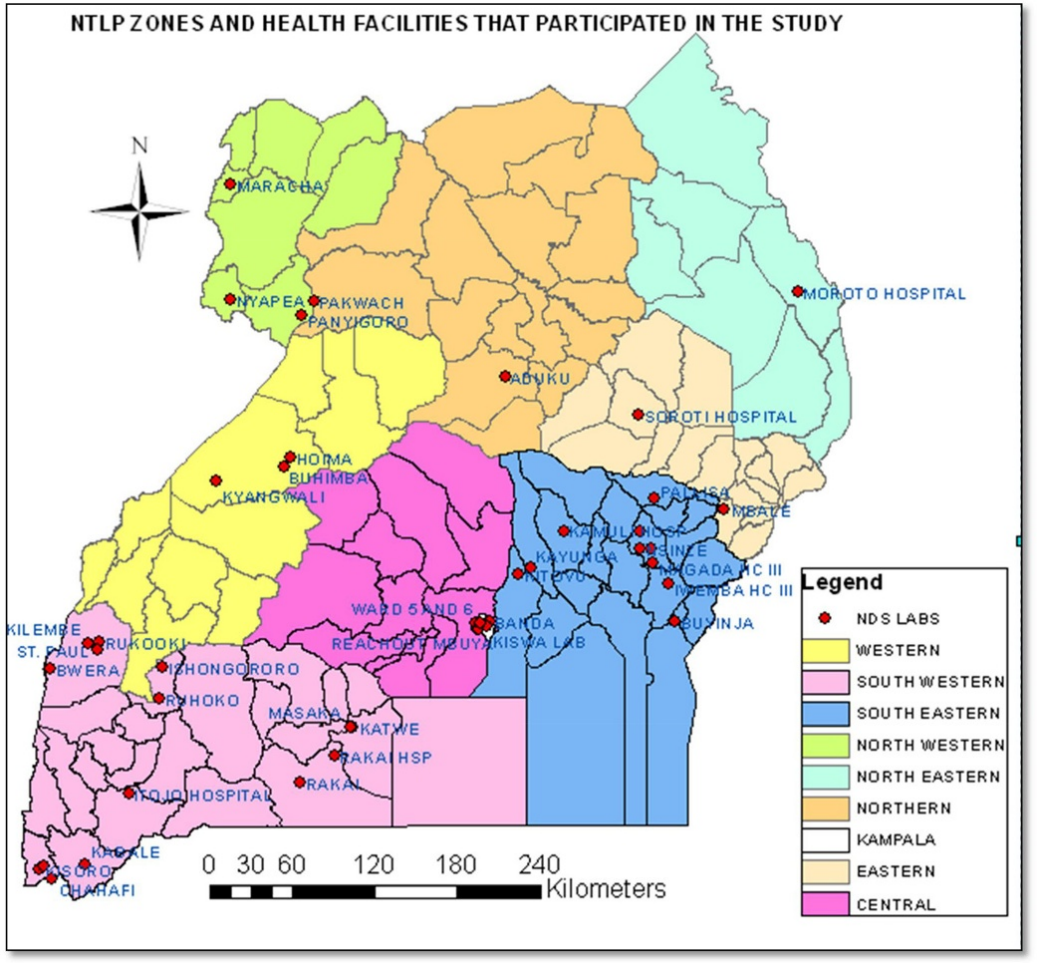
**Map of Uganda showing health facilities which participated in the study and the NTLP zones.**

The study included new and previously treated sputum smear-positive TB patients enrolled in a national anti-TB drug resistance survey from all the NTLP zones excluding the Central Zone (see Figure 1). This was a cross-sectional survey that included 44 TB diagnostic and treatment units across the nine TB control zones (DTUs) in the country (Figure 1). A cluster sampling method was used in which 30 clusters (primary sampling units) were selected randomly with probability proportional to the number of smear-positive TB patients registered in 2007. Within each cluster a fixed number of consecutively diagnosed smear-positive patients were enrolled so that all included patients had identical sampling probabilities. Each cluster was required to enrol 50 new sputum smear-positive TB patients (for a target sample size of 1500 patients) within one year, plus all the previously treated patients diagnosed during this period.

### Laboratory methods

#### Sputum collection

Each eligible patient who consented provided two sputum samples, an early morning and a spot sample, independent of the routine samples used for diagnostic purposes to minimize chances of contamination. Samples were refrigerated at 4°C and then transported to the National TB Reference Laboratory (NTRL) for processing via a local courier system.

#### Sputum culture and drug susceptibility testing

At the NTRL, samples were decontaminated using the 1.5% NaOH NALC method and processed. The sample was inoculated on two slopes of Löwenstein-Jensen (L-J) medium, incubated at a temperature of 37°C and monitored weekly for growth up to 8 weeks. A culture was only reported negative if no growth was shown after 8 weeks. For the positive cultures identification of MTB was done based on presumptive phenotypic appearance of colonies on the medium, and confirmed using insertion sequence 6110-based PCR method as previously described [23].

Isolates were tested for resistance to rifampicin, isoniazid ethambutol and streptomycin using the L-J proportional method, in concentrations of 40 μg/ml for rifampicin, 0.2 μg/ml for isoniazid, 2.0 μg/ml for ethambutol and 4.0 μg/ml for streptomycin, and all identified MDR-TB isolates were tested for resistance to kanamycin and ofloxacin in concentrations of 30 μg/ml and 2.0 μg/ml, respectively, using the method as previously described [24]. All samples resistant to rifampicin, a random sample of 20 isolates from previously treated patients susceptible to isoniazid and rifampicin, and a random sample of isoniazid resistant isolates that were susceptible to isoniazid were retested by the supranational reference laboratory in Borstel (Germany) for external quality assurance.

#### Preparation of crude genomic DNA from Mycobacterium tuberculosis for use as template in PCRs

A total of 1039 isolates were stored in replicates at the (NTRL) at −80°C. To extract DNA, the selected isolates were thawed overnight at – 20°C and later at room temperature for 12 hours. The vials were centrifuged at 15,000 g for 30 min and the pellet was washed twice with 500 ul of Qiagen PCR water. The final pellet was re-suspended in 100 μl of Qiagen PCR-water, heated at 95°C for 30 minutes to kill and lyse the bacilli and later sonicated for 15 min at room temperature. The extracted crude genomic DNA in the supernatant was recovered by centrifugation at 15,000 g for 30 min; the latter was used immediately in the real time PCR (RT-PCR) assay or stored at −20°C for future use.

#### SNP typing of *Mycobacterium tuberculosis*

A SNP (Rv0040c-0619n) identifying the Uganda II (MTB) with its accompanying designed primers and probes as previously described [18] was used in a real-time PCR [(RT)PCR] SNP assay. The (RT)PCR analysis involved 2 steps. The first was amplification (40 cycles of 95°C for 10 sec, 57°C for 10 sec and 72°C for 10 sec of the target region(s)) to generate amplicons for the melting curve analysis. The results of melting curves were analyzed using LightCycler® software version 1.5 to assign an isolate to a particular lineage depending on the melting temperature at which the hybridization probes dissociates from the amplicons. In all the assays we used MTB Uganda (MTB L4-U) genomic DNA from our laboratory, H37Rv genomic DNA (MTB lineage 4) and Central Asian strain (lineage 3) genomic DNA as control DNA.

#### HIV testing

HIV testing at all the 44 health care facilities followed the national algorithm using Unigold® (Trinity Biotech, Wiclock, Ireland) and Determine® (Abbot, Tokyo, Japan) as the screening and confirmatory tests respectively. A third kit, Stat-Pak® (Chethambutolio Medford NY, USA) was used when a mismatch between the confirmatory and the screening HIV test results on the same sample were reported. All participants were offered this test algorithm in accordance with the Uganda Ministry of Health guidelines.

### Data management and analysis

Data on demographics, microscopy, culture and DST results was double entered in epi-info V6 (2000 CDC Atlanta GA USA), as part of the anti-TB drug resistance study, while results on molecular studies was entered in MS Excel. Analysis was done in Stata v10 (STATA Corp. College Station TX USA.) The Chi-square or the 2-sided Fisher’s exact tests were used where appropriate for comparison of categorical variables. Univariate and multivariate analysis was done using logistic regression to identify variables associated with the T2/Uganda II MTB genotype. Contribution of the variables to the model was done using the likelihood ratio Chi square test. All significance testing was at the 95% confidence level.

Our primary outcome was the proportion of MTB patients with the Uganda II family calculated as a proportion across all clusters after weighing for the exact sampling probabilities for each new individual patient for whom DST results were available. Associations with MDR, HIV infection, patient age, previous history of TB treatment and urban or rural residence were also studied. In all these calculations, confidence intervals and p-values were adjusted for cluster design by first order Taylor linearization and by second order correlation of Rao and Scott of the Pearson *X*^2^ respectively as implemented by Stata svy commands [25].

### Ethical approval and consent procedure

Ethical and scientific approval of the study was granted by Makerere University College of Health Sciences, Faculty of Medicine review board and the Uganda National Council of Science and technology. All adult participants gave written informed consent before participation. Additionally, we obtained written informed consent from the parent/guardian of participants under the age of 18 years. The consent process included storage and use of the collected sputum samples for further studies.

## Results

Out of the 1537 patients included in the survey, 127 were culture negative, 77 samples were contaminated, eight samples grew *non-tuberculous mycobacteria,* and 1325 samples grew MTB based on IS6110-PCR analysis. Of these 1325 isolates, 324 did not grow on subculture (done for molecular analysis), leaving 1001 available for genotyping. Twenty-eight of these were excluded from further analysis due to inconclusive genotyping results. Thus, results on MTB genotype were available for single isolates of 973 patients. Of these isolates, 630 (64.8%) belonged to male participants. Mean age of participants was 34.7 years, 25–34 years being the modal age group (n = 330; 34.3%). Out of the eight zones that participated in this study, *south west* zone contributed the highest number of isolates (n = 313; 32.0%). Of the 973 patients, 297 (30.5%) tested positive for HIV, 217 (22.3%) of whom had already tested HIV positive before enrolment. Previously treated TB patients contributed 78 (8.1%) of the isolates. Generally there was no difference with regard to demographic characteristics (with exception of the zone where the patient was enrolled) of the patients included in the survey between those whose isolates were included and those whose were not included in the molecular typing (Table 1).

**Table 1:** **Characteristics of patients enrolled in the study**

Characteristic		Enrolled in survey N = 1537 n (%)	Included for molecular analysis N = 973 n (%)	Not included for molecular analysis N = 564 n (%)	p-value
**Sex**	Male	1018 (66.2)	630 (64.7)	388 (68.8)	0.106
	Female	519 (33.8)	343 (35.3)	176 (31.2)	
**Age**	13-14	33 (2.2)	21 (2.2)	12 (2.1)	0.956
	15-24	299 (19.5)	189 (19.8)	108(19.2)	
	25-34	521 (33.4)	330 (34.3)	191 (34.0)	
	35-44	366 (23.8)	235 (24.5)	131 (23.3)	
	45-54	167 (10.9)	100 (10.4)	67 (11.9)	
	>55	139 (9.0)	86 (9.0)	53 (9.43)	
**HIV already diagnosed positive**	Yes	339 (22.1)	217 (22.3)	122 (21.6)	0.586
	No	873 (53.6)	527 (54.2)	296 (52.5)	
	Unknown	375 (24.4)	229 (23.5)	146 (25.9)	
**TB control Zone**	South West	482 (31.4)	278 (28.6)	204 (36.2)	0.004
	Kampala	457 (29.7)	313 (31.2)	144 (25.5)	
	South East	97 (6.3)	59 (6.1)	38 (6.7)	
	Eastern	53 (3.5)	28 (2.9)	25 (4.4)	
	North East	152 (9.9)	89 (9.2)	63 (11.2)	
	North	52 (3.4)	36 (3.7)	16 (2.8)	
	North West	149 (9.7)	104 (10.7)	45 (8.0)	
	Western	95 (6.2)	66 (6.8)	29 (5.1)	
**HIV results at (re)testing**	Positive	469 (30.5)	297 (30.5)	172 (30.5)	0.216
	Negative	1054 (68.6)	664 (68.2)	390 (69.2)	
	Missing	14 (0.91)	12 (1.23)	2 (0.35)	
**Previous TB treatment**	Yes	140 (9.2)	78 (8.1)	62 (11.3)	0.057
	No	1397 (90.8)	883 (91.9)	500 (89.0)	

Of the 973 MTB isolates analysed with valid results, 422 (43.4%) were of the Uganda II family. Among the eight NTLP zones that participated in this survey, *south west* zone had the highest proportion of this genotype, 172/313 (55.0%; 95% CI, 49.2-60.5), while *south east* had the lowest 7/35 (19.4%; 95% CI 8.1-36.0). We found the Uganda II family less distributed in the *east* and *south east* zones as compared to the *south west* zone (p < 0.001) (Table 2). The 25–34 year age group had the highest proportion of the Uganda II family (48.2%) although there was no statistical significance of the differences in distribution of this genotype among the different age groups. We found no association between the Uganda II family distribution with previous history of TB treatment and rural/urban residence.

**Table 2:** **Analysis of risk factors associated with Uganda II family among sputum smear-positive TB patients in Uganda**

Risk Factor		Proportion n/N (%) N = 961	** Univariate analysis	
			OR (95% CI)	p-value
Sex	Male	279/630 (44.3)	1.12 (0.83-1.52)	0.420
	Female	143/343 (41.7)	Ref.	
Age	15-24	71/189 (37.6)	Ref.	
	13-14	10/21 (47.6)	0.66 (0.25-1.72)	0.386
	25-34	159/330 (48.2)	0.65 (0.45-9.20)	0.017
	35-44	96/235 (40.9)	0.87 (0.62-1.21)	0.406
	45-54	45/100 (45.0)	0.74 (0.46- 1.19)	0.210
	>55	35/86 (40.7)	0.87 (0.47-1.60)	0.660
Zone	South West	172/313 (55.0)	Ref.	
	Kampala	112/278(40.3)	0.55 (0.36-0.83)	0.006
	Eastern	22/89 (24.7)	0.26 (0.17-0.41)	<0.001
	South East	7/36 (19.40)	0.20 (0.14-0.28)	<0.001
	North East	26/59 (44.1)	0.64 (0.35-1.17)	0.146
	North	10/28 (35.7)	0.45 (0.31-0.64)	<0.001
	North West	20/66 (30.3)	0.32 (0.18-0.55)	<0.001
	Western	53/104 (51.0)	0.86 (0.43-1.72)	0.667
Previously treated for TB	Yes	31/78 (39.7)	0.85 (0.58-1.25)	0.403
HIV status	Positive	130/442 (29.4)	1.09(0.73-1.63)	0.648
Diagnosis made in an urban setting	Yes	267/585 (45.6)	0.80 (0.49-1.32)	0.385

** Odds ratios and confidence intervals adjusted for cluster design.

On multivariable analysis, isolates belonging to other genotypes other than the Uganda II family were more likely to have resistance to any of the first-line drugs (OR^adj^ 2.9; 95% CI, 1.63-5.06; p = 0.001) and more likely to be MDR (OR^adj^ 4.9; 95% CI, 1.15-20.60; p = 0.032) than the Uganda II family, even though MDR isolates were few (n = 23). Included in our model for adjustment were the NTLP zone where the patient was enrolled, patient category (new or previously treated), age group, and participant residence (rural/urban) (Table 3). In NTLP zones where the Uganda II family was less distributed, participants had an increased risk of up almost twice (OR^adj^ 1.8; 95% CI, 1.25-2.62; p = 0.001) of having drug resistance as compared to *south west* zone where this genotype contributed a larger proportion to the sample (Table 4). No association between HIV infection and the Uganda II family (OR 1.09; 95% CI, 0.73-1.63; p = 0.648) was established at univariable analysis.

**Table 3:** **Multivariate analysis of Uganda II family and anti-TB drug resistance among sputum smear-positive TB patients in Uganda**

Genotype	Resistance Pattern							
	Any resistance				MDR			
	Proportion	**OR	95% CI	p-value	Proportion	**OR	95% CI	p-value
**Uganda II**	26/422 (6.2)	Ref.			3/422 (0.7)	Ref.		
**Other**	91/549 (16.6)	2.9	1.63-5.06	0.001	19/530 (3.5)	4.9	1.15-20.60	0.032

Others included in the model were National TB/Leprosy Program (NTLP) zone, patient category (new or previously treated), HIV status, age group, and residence (rural/urban).

** Odds ratios adjusted for cluster design.

**Table 4:** **Multivariable analysis of ‘any resistance’ and ‘NTLP zone’ in relation to the proportion of the Uganda II family among the sputum-smear positive TB patients in Uganda**

	Total number of isolates N = 973	Proportion of T2 isolates n (%)	Univariate analysis		Multivariable analysis	
Zone			**OR (95% CI)	p-value	OR (95% CI)	*p-value
**South West**	313	172 (55.0)	Ref.		Ref.	
**Kampala**	278	112 (40.3)	1.9 (1.34-2.79)	0.004	1.8 (1.23-2.53)	0.005
**Eastern**	89	22 (23.6)	1.9 (0.76-4.79)	1.57	1.9 (0.8-4.43)	0.122
**South East**	36	7 (19.4)	1.7 (1.26-2.35)	0.001	1.8 (1.25-2.62)	0.003
**North East**	59	28 (44.1)	1.9 (1.27-2.73)	0.030	1.8 (1.23-2.53)	0.001
**North**	28	10 (35.7)	0.8 (0.58-1.08)	0.137	1.0 (0.69-1.45)	0.980
**North West**	66	20 (30.3)	0.5 (0.27-1.10)	0.295	0.73 (2.05-2.78)	0.113
**Western**	104	53 (51.0)	1.5 (0.22-1.61)	0.134	1.6 (0.89-2.78)	0.546

** Odds ratios confidence intervals and p-values adjusted for cluster design.

* = P value for the difference in resistance between the *south western* zone and each of the other zones included in the study.

Other variables included in the model are patient category, sex, residence (rural/urban), HIV status.

Ref = reference category.

## Discussion

In this cross-sectional molecular analysis of *M. tuberculosis* strains circulating in Uganda, we clearly demonstrate the Uganda II family as the major cause of morbidity due to TB in the country although at different levels of predominance in the eight NTLP (i.e. geographic) zones. Its frequency was highest in the *south west* and lowest in the *south east* zone (see Figure 1). Our study showed that this genotype is less likely to develop resistance to any of the anti-TB drugs compared to all the other genotypes taken as a single group, but no association with HIV infection was demonstrated.

We found the Uganda II family about 2.9 times less likely to be resistant to any of the first-line anti-TB drugs and almost 5 times less likely to be MDR as compared to the non Uganda II MTB family. It confirms our observation from a similar survey done in Kampala where the T2 Uganda genotype strains were less likely to be drug resistant compared to other, geographically more diverse genotypes such LAM, CAS and T1 [22]. That the Uganda II family is negatively associated with M/DR-TB is further supported by the higher levels M/DR-TB in zones where this genotype is less prevalent such as the *east* zone. Here the prevalence of drug resistance was up to almost 2 times higher than in the *south west* zone where the majority of the isolates belonged to the Uganda II family after adjusting for patients’ HIV status, treatment category, rural or urban residence and sex (Table 4). The negative association reported in our earlier analysis [22] between the T2 Uganda genotype and any drug resistance is thus confirmed in this study, and also exists for MDR (stronger than even the association with any resistance), possibly due to larger numbers of MDR-TB patients to establish a statistically significant difference.

Contrary to our findings, in some high burden TB settings the predominant MTB genotype has been associated with M/DR. The Beijing genotype in particular has been documented to cause outbreaks of MDR-TB where this strain is the major cause of TB morbidity [26]. It remains a subject for further analysis whether the Uganda II family is inherently less likely to become drug-resistant (i.e. has a lower potential for acquiring drug resistance), or that drug-resistant Uganda II family strains are less easily transmitted than other drug-resistant strains. The latter could occur if for example (multi)drug-resistant Uganda II strains cause clinical infections with lower bacterial load, or show higher cure rates with first-line treatment, than do (multi)drug-resistant strains of other genotypes. This could reflect differences in adaptation to the Ugandan host population between the more indigenous Uganda II family strains and more recently imported strains such as LAM, CAS and T1. In addition, one could speculate that predominance of the Uganda II family plays a causative role with regard to reported low levels anti-TB drug resistance in Uganda [5], and that similar genotypic differences in association with drug resistance exist elsewhere in sub-Saharan Africa where low levels of (M)DR-TB have been reported.

Despite a high TB/HIV co-infection rate in our sample (30.2%) we found no association between HIV infection and Uganda II strains similar to findings of related studies elsewhere in SSA [27]. This finding suggests that the predominance of the Uganda II strains is unlikely to be due to changes in immunological pressure on the circulating MTB population as a result of the HIV epidemic.

Since we observed significant geographical variations with respect to the distribution of the Uganda II family, it can be speculated that the *north east* and *south east zones* where this genotype is less distributed might have a diversity of MTB strains that are imported through cross-border movement with neighbouring countries such as Kenya, Tanzania and beyond. Indeed the eastern border of Uganda acts as the entry/exit (Busia and Malaba) for goods and people from Mombasa port and the northern part of Kenya and, the Kilimanjaro region in Tanzania, while Kampala being the capital city is the major business centre and a pathway for people entering and exiting Uganda from all over the world. Human traffic at these entry/exit points might enhance transmission of communicable diseases, including TB, with higher probability of strain diversity than in the *south west* zone. The other possibility could be that the Uganda II family might be genetically more adapted to indigenous human populations of the *south west* than the *east* and *south east* zones. Both hypotheses can only be confirmed through further research.

### Limitations

Our study had limitations. We did not fully characterise the non-Uganda II family into specific lineages that have been previously documented as significantly contributing to the TB burden in this setting. Therefore we could not fully conclude which particular strain drives drug resistance in this locality and any associated factors such as transmissibility and potential to develop drug resistance as documented elsewhere. However our aim was to find out the role of the predominant genotype in the observed low levels of anti-TB drug resistance in this locality and our earlier report [22], though limited in terms of geographical coverage, fully characterised the less predominant MTB lineages in relation M/DR-TB and HIV infection. We however strongly recommend a more in depth analysis to characterize the ‘non- Uganda II family’ and its distribution throughout Uganda. Secondly, we employed a typing method (identification of genotype-specific SNPs) that was different from the method that was used for the Kampala survey (spoligotyping) in which the negative association of the T2 Uganda genotype with drug resistance was initially observed. However, earlier studies have shown that the T2 genotype and the Uganda II genotype strongly overlap [18]. Thirdly, only sputum smear-positive TB patients were included in the study since this was an extension of a drug resistance survey. This sampling frame could limit generalizability of our results to smear negatives should there be a difference in the distribution of the Uganda II family strains. Since differences in bacterial load and resulting transmission fitness of drug resistant strains may in part explain the observed association, it would be interesting to extend the current study with sputum smear-negative patients.

Few MDR patients were available to provide enough power for analysis of associations between the Uganda II family and MDR although we managed to demonstrate this with wide confidence interval.

## Conclusion

This study shows the Uganda II family to be the predominant MTB genotype in Uganda and confirms earlier findings that this genotype is less likely to be drug resistant compared to other genotypes combined. This may in part explain the low levels of drug resistance found in Uganda. Similar negative associations between the predominant indigenous MTB strains and drug resistance may exist elsewhere in SSA [28].

## Authors’ original submitted files for images

Below are the links to the authors’ original submitted files for images. Authors’ original file for figure 1
