# Supplementary figures and images for: The Mycobacterium tuberculosisUganda II family and resistance to first-line anti-tuberculosis drugs in Uganda
Source: BMC Infect Dis. 2014 Dec 19;14:703. doi: 10.1186/s12879-014-0703-0 (PMC4367914; doi:10.1186/s12879-014-0703-0)

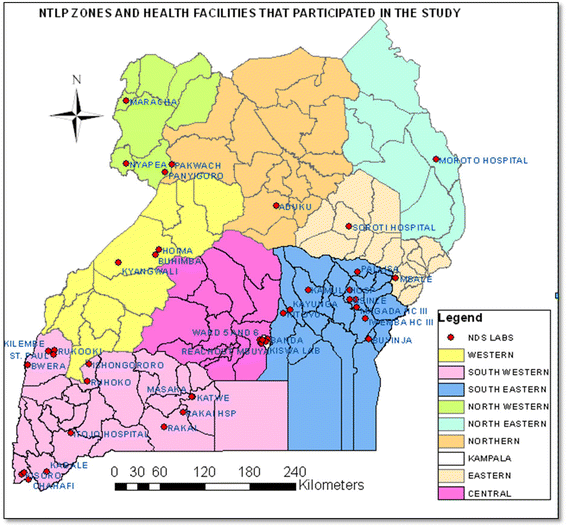

Supplement: Supplementary file 1 — Authors’ original file for figure 1 [file 12879_2014_703_MOESM1_ESM.gif]
